# Perfect date—the review of current research into molecular bases of mammalian fertilization

**DOI:** 10.1007/s10815-019-01679-4

**Published:** 2020-01-06

**Authors:** Zuzana Trebichalská, Zuzana Holubcová

**Affiliations:** 1 Faculty of Medicine, Department of Histology and Embryology, Masaryk University, Kamenice 5, Brno, Czech Republic; 2 Reprofit International, Clinic of Reproductive Medicine, Brno, Czech Republic

**Keywords:** Fertilization, In vitro fertilization, Gamete biology, Human reproduction

## Abstract

Fertilization is a multistep process during which two terminally differentiated haploid cells, an egg and a sperm, combine to produce a totipotent diploid zygote. In the early 1950s, it became possible to fertilize mammalian eggs in vitro and study the sequence of cellular and molecular events leading to embryo development. Despite all the achievements of assisted reproduction in the last four decades, remarkably little is known about the molecular aspects of human conception. Current fertility research in animal models is casting more light on the complexity of the process all our lives start with. This review article provides an update on the investigation of mammalian fertilization and highlights the practical implications of scientific discoveries in the context of human reproduction and reproductive medicine.

## Introduction

Reproduction ensures the maintenance of a population and the evolution of all species. The ability to produce viable gametes, along with mating strategies resulting in encounters and the union of these highly differentiated cells, is essential for all mammals. Although the fertilization process is central for the reproduction of our species, its molecular bases remain poorly understood. Since experimentation on humans is hindered by ethical, technical, and regulatory challenges, the animal models are employed to explore fundamental principles of sperm-egg interaction. Here, we recapitulate current knowledge of the mammalian fertilization, especially focusing on newly identified molecular players involved in (1) oocyte-induced sperm hyperactivation, (2) mutual gamete recognition and binding, (3) oocyte activation, and (4) prevention of polyspermy. The published research findings are critically discussed in relation to their role in human fertility treatment, reproductive toxicity, and birth control. The last section is dedicated to future directions in the field of fertility research and reproductive medicine.

### Oocyte-induced sperm hyperactivation

Only high-quality gametes that meet stringent selection criteria can conceive a new life. Very few of the millions of ejaculated spermatozoa are selected by the female reproductive tract to gain access to the fertilization site. On their epic journey, male gametes have to use their limited resources and overcome multiple mechanical and chemical impediments [1]. The interaction with fluids secreted by the female reproductive system facilitates sperm functional maturation. This process called “capacitation” involves complex modification in the molecular landscape of the sperm plasma membrane. Exposure of the membrane receptors is essential for the spermatozoon to acquire the full capacity to interact with the ovulated egg. Molecular details of the capacitation process have been comprehensively reviewed elsewhere and are not covered here [2, 3]. Simultaneously, multiple external cues alter sperm motility and swimming patterns. Biochemical signals present in the female reproductive tract cause a shift of flagellar movement from a low amplitude and regular symmetric beat, into a short asymmetric pounding. Such a vigorous type of motility is believed to generate a more powerful force which enhances release of sperm from the oviductal reservoirs, their efficient movement in viscoelastic luminal fluids, invasion of the cumulus oophorus, and ultimately penetration of the glycoprotein-rich mesh of zona pellucida (ZP) [4, 5]. The transformation of the sperm motility pattern is called “hyperactivation” and, along with capacitation, renders the sperm competent to fertilize the ovum.

There is a plethora of regulatory pathways that control the physiology of human spermatozoa. Among them, the elevation of intracellular calcium (Ca^2+^) is a key signaling element implicated in sperm maturation, motility, chemotaxis, and acrosome reaction, all of which are critical for the success of fertilization. Free intracellular Ca^2+^ ions exert instant allosteric regulatory effects on enzymes and proteins and act as a second messenger ensuring the transduction of the information from membrane receptors to downstream effector molecules. Animal studies using a demembranated sperm model showed that Ca^2+^ acts directly on cytoskeletal components of the flagellum to regulate sperm motility [6, 7]. The change from basal to hyperactivated motility is triggered by a rapid rise of intracellular concentration of Ca^2+^ ([Ca^2+^]i) resulting from an influx of Ca^2+^ across the sperm plasma membrane. The experimental data obtained from the application of “patch-clamp” recordings have revealed the central role of the CatSper channel in Ca^2+^ signaling.

#### CatSper: unique Ca2+ channel of sperm flagellum

The CatSper is a weakly voltage-gated and pH-sensitive ion channel with a high affinity to divalent cations [8, 9]. This multi-protein complex is composed of four pore-forming subunits CatSper1–4 and five accessory subunits encoded by at least nine genes in mammals [10, 11]. The structure and distribution of its subunits are integral for the channel function [11–13]. The expression of CatSper proteins is testis-specific with localization confined to the surface of the sperm tail [14]. Genetic evidence shows that the function of CatSper channel proteins is indispensable for male fertility. The knockout mice were sterile despite having normal sperm count and morphology. Their spermatozoa exhibited impaired motility and failed to penetrate ZP [12]. In humans, genetic lesions and altered expression profiles of CatSper genes have been clinically linked to asthenoteratospermia and male infertility [15–19].

**Fig. 1:**
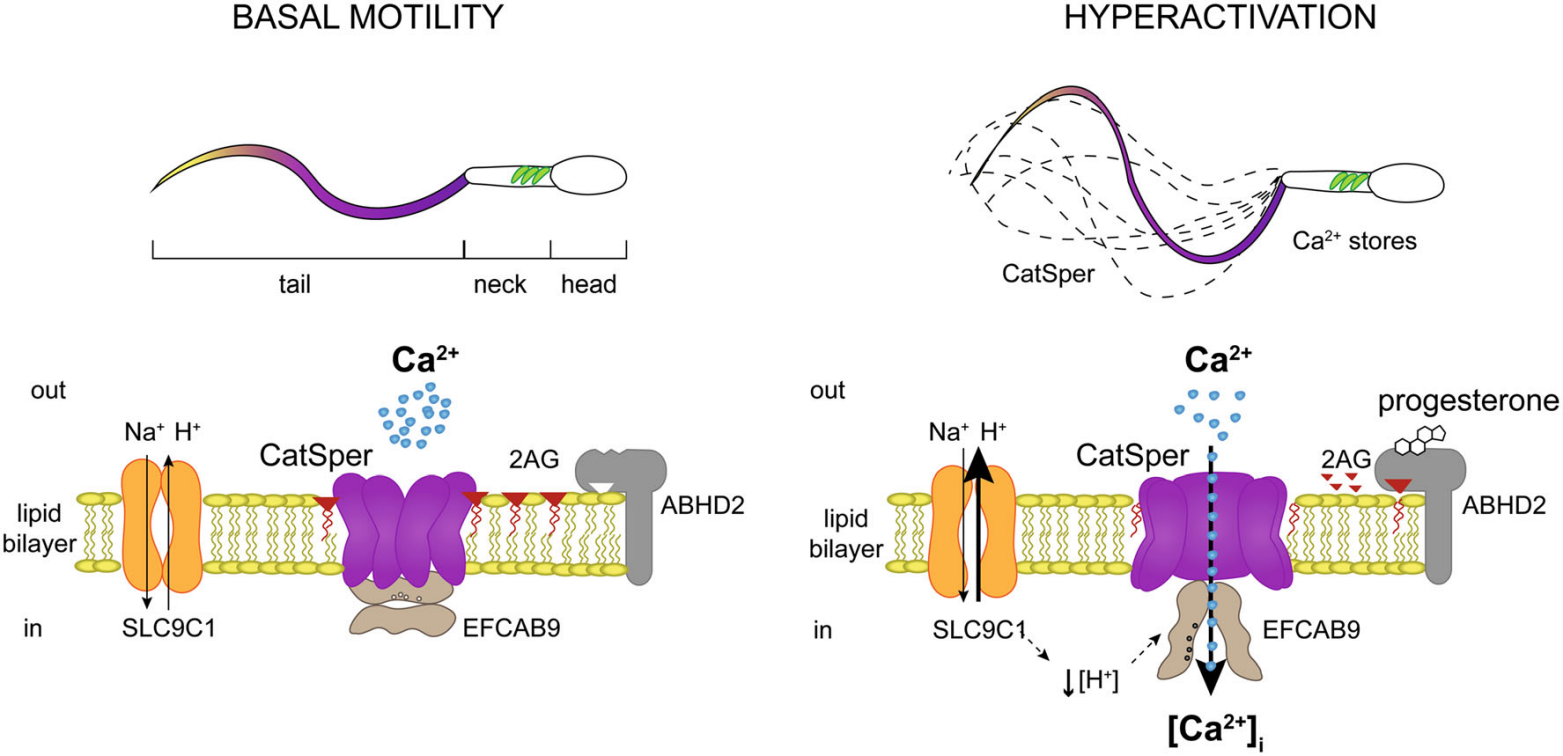
The role of Ca^2+^ signaling in sperm hyperactivation. Progesterone-induced activation of the CatSper channel in an alkaline environment allows rapid Ca^2+^ influx which, along with mobilization of Ca^2+^ from the storage organelles, elevates intracellular Ca^2+^, thus generating the hyperactivated sperm motility

To achieve fertilization, hyperactivated motility must be switched on at the right place and time. The ability of Ca^2+^ to evoke asymmetric flagellar beating is known to be primed by increased pH [20]. But how the capacitation-induced rise of intracellular pH regulates Ca^2+^ influx was unclear. Recently, SLC9C1, a sperm-specific Na/H^+^ exchanger mediating chemoattractant-induced intracellular alkalization, has been identified as a gatekeeper at the pore of the CatSper channel [21]. The elevation of intracellular Ca^2+^ in the sperm tail induces a conformational change of the blocker molecule. Subsequent dissociation of the SLC9C1 opens the CatSper channel for the Ca^2+^ entry [22] (Fig. 1). This finding provides molecular explanation for CatSper sensitivity to pH. In vivo, oviduct lumen pH increases after ovulation thus providing favorable conditions for sperm hyperactivation [23]. Yet, some researchers intuitively searched for a more specific physiological stimuli produced by the female reproductive tract.

Two simultaneously published studies revealed an unprecedented role of sex hormone progesterone as a hyperactivation-inducing factor [24, 25]. This female steroid hormone, secreted by cumulus cells that surround the oocyte after ovulation, is a well-known chemoattractant for mammalian spermatozoa [26]. The nanomolar concentration of progesterone has been shown to dramatically potentiate hyperactivated flagellar movement of human spermatozoa in an alkaline environment. Surprisingly, progesterone-induced hyperactivation does not involve a slow canonical pathway of interaction with nuclear receptor and alteration of gene expression. Instead, the steroid hormone binds to a membrane receptor on the extracellular side of the cell bringing about a CatSper-mediated influx of Ca^2+^. The non-genomic action of progesterone on CatSper is intermediated by α/β hydrolase domain-containing protein 2 (ABHD2) which serves as a progesterone receptor on the sperm membrane. Upon progesterone binding, lipid hydrolase ABHD2 degrades endocannabinoid 2-arachidonoylglycerol (2AG) present on the sperm membrane. The replenishment of AG2 leads to the CatSper opening and triggers sperm hyperactivation (Fig. 1). Since ejaculated human spermatozoa retain a substantial amount of 2AG, progesterone-stimulated removal of 2AG by ABHD2 is required for full activation of CatSper [27]. Similar to progesterone, prostaglandin E1 (PGE1) stimulates the activity of human CatSper, but apparently through a different binding site [24]. Structural similarity of PGE1 with a 2AG tail implies that there might be direct competition between PGE1 and 2AG [27]. Further research is needed to test this hypothesis and provide a robust insight into how PGE1 regulates the function of the CatSper channel. Although both mouse and human CatSper are pH-sensitive, mouse CatSper is not activated by progesterone or prostaglandins [24]. The interspecies differences in expression and function of this ion channel can be attributed to the low degree of sequence identity between orthologs of CatSper units [28]. For this reason, mice “knockout” models cannot be used to investigate and progesterone/PGE1-mediated hyperactivation pathway.

The ligand-sensitive nature of human CatSper activation predisposes human sperms to be modulated by various compounds which bind to the extracellularly accessible site of this channel complex. Physiological stimuli which have been recognized to induce CatSper-dependent Ca^2+^ entry include glycoproteins of ZP, serum albumin, and cyclic nucleotides [29, 30]. The predominant expression in sperm makes the CatSper channel an attractive target for modulation of male fertility [31–33]. For instance, steroid-like plant terpenoids, lupeol and pristimentin, were identified as potent inhibitors of hyperactivation of capacitated spermatozoa. Both molecules compete with progesterone for its binding site on ABHD2 and can thus act as contraceptive compounds. Interestingly, both testosterone and hydrocortisone were found to antagonize the action of progesterone at physiological concentrations, which may explain why elevated levels of these steroids in the female organism adversely affect fertility [34]. Importantly, a range of structurally diverse molecules including environmental pollutants, endocrine disruptors, odorants, and common drugs have been shown to interact with the CatSper and interfere with hyperactivation signaling (Table 1) [30, 35–41]. These exogenous chemicals desensitize spermatozoa to physiological CatSper agonists and thus may impair their functional competence. Future studies that would determine safety thresholds for acute and chronic exposure to these substances for menʼs reproductive health are urgently needed. Taken together, the CatSper channel serves as a polymodal sensor that integrates various chemical clues from the surrounding microenvironment and translates them into Ca^2+^ signaling patterns, which, in turn, modulates the amplitude of flagellar beating. The complex regulation of motility might be functionally important for navigation of sperm over long distances to reach the site of fertilization.

**Table 1:** Overview of the identified low molecular interactors of the CatSper channel

Type	Category	Name	CAS number	Concentration	Effect	Reference
endogenous	steroids	progesterone	57-83-0	nM	agonist	*Lishko et al., 2011 *[24]
						*Strunker et al., 2011 *[25]
		testosterone	58-22-0	nM	partial agonist	*Mannowetz et al., 2017 *[34]
		estrogen	50-28-2	nM	partial agonist	*Mannowetz et al., 2017 *[34]
		hydrocortisone	50-23-7	nM	partial agonist	*Mannowetz et al., 2017 *[34]
	endocannabidoids	2-arachidonoylglycerol	53847-30-6	μM	antagonist	*Miller et al., 2016 *[27]
exogenous	analogues of cyclic nucleotide	8-bromoguanosine 3’,5’-cyclic monophosphate	31356-94-2	mM	agonist	*Brenker et al., 2012 *[30]
	triterpenoids	pristimerin	1258-84-0	nM	partial agonist	*Mannowetz et al., 2017 *[34]
		lupeol	545-47-1	nM	partial agonist	*Mannowetz et al., 2017 *[34]
	nonsteroidal estrogens	α-zearalenol	36455-72-8	μM	agonist	*Brenker et al., 2018 *[41]
						*Schiffer et al., 2014 *[36]
	endocrine disruptor	dichlorodiphenyldichloroethylene	72-55-9	pM - μM	agonist	*Tavares et al., 2013 *[35]
		diethylstilbestrol	56-53-1	pM - μM	agonist	*Zou et al., 2017 *[40]
	plasticizers	dibutyl phtalate	84-74-2	μM	agonist	*Schiffer et al., 2014 *[36]
	anesthetic	ketamine	6740-88-1	mM	antagonist	*He et al., 2016* [38]
	odorants	bourgeonal	18127-01-0	μM	agonist	*Brenker et al., 2012 *[30]
		undecanal	112-44-7	μM	agonist	*Brenker et al., 2012 *[30]
		cyclamal	103-95-7	μM	agonist	*Brenker et al., 2012 *[30]
		helional	1205-17-0	μM	agonist	*Brenker et al., 2012 *[30]
	UV filters	benzylidene camphor sulfonic acid	56039-58-8	μM	agonist	*Brenker et al., 2018 *[41]
		4-methylbenzylidene camphor	36861-47-9	μM	agonist	*Rehfeld et al., 2016 *[37]
		methyl anthranilate	134-20-3	μM	agonist	*Rehfeld et al., 2016 *[37]
		isoamyl p-methoxycinnamate	71617-10-2	μM	agonist	*Rehfeld et al., 2016 *[37]

The vital role of CatSper-mediated influx of Ca^2+^ from the extracellular fluid is generally recognized. But there is also a piece of good evidence for the functional significance of Ca^2+^ stored in intracellular organelles. Unlike most cells, mature spermatozoa do not contain endoplasmic reticulum, which is a primary Ca^2+^ storage organelle. Potential areas for functional Ca^2+^ store in sperm include acrosome, mitochondria in the midpiece and redundant nuclear envelope in the connecting piece [42, 43]. The exact mechanism of Ca^2+^ mobilization from internal reservoirs remains to be elucidated. In the tentative model, CatSper-mediated elevation of flagellar [Ca^2+^]i spreads forward and stimulates the secondary release of stored Ca^2+^ thus leading to hyperactivation [43, 44]. Apart from the conventional store-mobilizing agonists, vitamin D or peptide hormones kisspeptin and ghrelin might be involved in the mobilization of Ca^2+^ in hyperactivated human sperm [43]. Although the evidence is preliminary, it is tempting to speculate that pharmacological activation of stored Ca^2+^ release might help to bypass the adverse effects on motility caused by impaired function of the CatSper.

### Sperm-egg association

The union of an egg with a sperm is a central event of the fertilization process. It involves the stages of the initial approach, molecular recognition, membrane apposition, and the merger of the plasma membrane lipids [45, 46]. To get into physical contact with the oolemma, the spermatozoa have to actively invade the cumulus and bore through the matrix of the ZP (details of this fertilization step are reviewed elsewhere [47, 48]). Acrosome reaction results in exposure of a new set of surface antigens thus priming the sperm for fusion with the oolemma. After reaching perivitelline space, the spermatozoa adhere to membrane protrusions, known as microvilli which densely cover the surface of the oocyte. These actin-filled hair-like projections greatly enlarge the contact area and decrease the repulsion between the two juxtapositioned membranes [49]. In many animal species, sperms have a preferential entry point [50–52]. In mouse, rat, and hamster eggs, sperm do not fuse with the microvilli-free cortical area overlying the meiotic spindle [53, 54]. On the contrary, in sperm-receptive human oocytes, microvilli are uniformly distributed all over the oocyte surface [55] suggesting that, in humans, the sperm entry can occur anywhere on the oocyte surface. Nevertheless, this does not exclude the existence of plasma membrane microdomains which would facilitate sperm adherence and thus serve as fertilization “hot spots” on the surface of a human egg [56].

The molecular mechanisms underlying complex dialogue between sperm and egg have been widely investigated in the mouse model. In vitro fertilization experiments, immunoassays, and targeted gene disruption have been used to identify the principal receptors involved in gamete conjunction. However, the majority of putative match-making factors turned out to be nonessential for fertilization in vivo [47, 57]. To date, only three proteins, namely, CD9, Izumo, and Juno, were proven to be indispensable for gamete interaction.

#### CD9

*CD9* was the first gene identified to have a sex-specific fertility effect. This ubiquitous membrane protein is localized to the oolemma of fertilization-competent eggs and required for normal microvilli morphology and distribution [58–60]. The observation that the monoclonal anti-CD9 antibody potently inhibits sperm-egg binding in vitro [59] indicated that this surface protein is involved in gamete interaction. Three studies independently reported that female, not male, *CD9*-null mice exhibited a dramatic reduction of fecundity resulting from the specific failure of membrane fusion [60–62]. The details of molecular mechanism by which the loss of the CD9 function impairs fertilization remain to be elucidated. Based on the experiments assessing the relationship between CD9 presence and adhesion phenotypes, Jegou et al. proposed a model in which CD9-driven formation of adhesion sites facilitate the tight sperm-egg contact necessary for fusion to take place [63]. Interestingly, CD9-containing vesicles secreted by wild-type oocytes have been reported to restore the fusing ability of the CD9-deficient mouse eggs and translocate CD9-positive membrane fragments onto the sperm head before its attachment to the egg membrane [64, 65]. These findings support the role of CD9 as an organizer of membrane architecture and suggest that not only the oolemma but also sperm membrane rearrangement is required for gamete fusion.

#### Izumo

Izumo, named after the Japanese shrine dedicated to marriage, is the key fertilization element expressed on the sperm surface. The antigen was identified through the generation of a monoclonal antibody that effectively blocks fertilization. The importance of this molecule has been demonstrated by knockout experiments. *Izumo*−/− male mice are completely sterile despite normal mating behavior and sperm production. Mutant spermatozoa can penetrate the ZP but fail to fuse with the oocytes. However, when the fusion step was bypassed by intracytoplasmic sperm injection (ICSI), activation took place and the embryos developed to term [66]. Izumo is expressed on the inner acrosomal membrane and is thus undetectable on the surface of freshly ejaculated spermatozoa. At the time of acrosome reaction, Izumo becomes exposed and diffuses from the acrosomal cap to the equatorial segment of the sperm head where gamete fusion is initiated [66, 67]. Testis-specific serine kinase TSSK6 has been implicated in this translocation process. The inability to properly redistribute Izumo can explain the absence of fusion underlying sterility of *Tssk6*-null male mice [68, 69]. Izumo was found to be expressed also in human sperm [66, 70] but, to date, there is a paucity of clinical data supporting its involvement in etiology of idiopathic infertility [71, 72]. Although active immunization against Izumo causes contraceptive effect in female mice, the plausibility of targeting Izumo, or another sperm-specific protein, for development of vaccines for human contraception remains controversial and awaits further investigation [73–75]. The discovery of two fusion-related antigens, a sperm-specific Izumo and oocyte-expressed CD9, stimulated further research into the molecular basis of sperm-egg recognition [58–60, 64]. Although both Izumo and CD9 are enriched in the adhesion area, in vitro cell binding assays revealed that there is no physical interaction between the two molecules [76, 77]. Therefore, the existence of alternative Izumo-binding factor(s) on the eggʼs surface has been hypothesized [77].

#### Juno

In 2014, Bianchi and colleagues used the recombinant probe containing IZUMO domain to find its receptor on the oocyte plasma membrane. This experiment led to the discovery of the complementary receptor Juno which was named after the ancient Roman goddess of marriage and childbirth. The observation that *Juno*^−/−^ oocytes bind but do not incorporate the sperm head provided genetic evidence for the vital role of the Juno receptor in the fusion process [78]. In humans, the incidence of certain sequence variants of the Juno-encoding gene has been related to unexplained fertilization failure [79]. Analysis of the protein expression in unfertilized the metaphase II and in vitro matured human oocytes showed that Juno accumulates to the oolemma in the course of human oocyte maturation [80, 81]. Yet, fluorescent live imaging of good-quality oocytes is needed to characterize Juno dynamics prior to fertilization and explain the discrepancy between the localization patterns immunodetected in fixed and live human samples reported in this study. Of note, two recent studies reported that the exposure of the female mice to widespread environmental pollutants, melamine and benzo[a]pyrene, altered expression and localization of Juno and adversely effected mouse egg fertilizing capacity assessed in vitro [82, 83]. Future studies should address whether the similar risk of reproductive toxicity exists in humans.

The pairing of the sperm-specific protein Izumo and its oocyte-expressed counterpart Juno constitutes an initial step in gamete fusion. High-resolution imaging of ectopically expressed fluorescent proteins has been used to study receptor-ligand interaction in vitro. The results indicated that monomeric Izumo is recognized by Juno and then transferred to an unidentified egg receptor while generating robust intercellular adhesion [66, 77]. Yet, heterologous fusion assays demonstrated that the binding of Izumo and Juno facilitates membrane tethering but it is not sufficient to trigger the union of the two cells [76]. This indicates the requirement of additional factor(s) conferring adhesion complex fusogenic property. There are many open questions concerning the interplay of key molecular players. According to the current model, binding of Izumo and Juno induces accumulation of CD9 in the contact area and thereby promotes CD9-mediated clustering of the membrane proteins that participate in the assembly of putative fusion machinery [76, 78]. The current goal for researchers is to identify the crucial fusion-inducing component of this machinery.

The nature of Izumo-Juno interaction was found to be conserved in several mammalian species including humans [77]. Normally, the ZP establishes interspecies reproductive isolation. But at the absence of the protective layer, certain orthologues of Izumo and Juno proteins may directly interact allowing for cross-species gamete fusion in vitro*.* In particular, it has been demonstrated that mouse Izumo is capable of inducing adhesion with the human egg membrane [75]. Further, the recombinanat hamster Juno can bind human, mouse, and pig Izumo whereas human Izumo interacts with hamster but not mouse Juno [84]. This data provides a molecular explanation for a long-recognized ability of acrosome-reacted human sperms to penetrate zona-free hamster eggs. The phenomenon of heterologous fusion can be clinically exploited to assess the human sperm fertilizing capacity in vitro [85]. Due to the identification of surface proteins, vital for sperm-egg interaction, it may now be possible to develop better diagnostic assays, sperm selection techniques, and a new generation of contraceptives. It would be also interesting to investigate whether the altered expression of and/or misrecognition of key fertilization receptors contributes to human idiopathic infertility and unexplained fertilization failure.

### Egg activation

Following their fusion with sperm, mammalian oocytes undergo periodic changes in cytosolic Ca^2+^ concentration, referred to as “calcium oscillations.” Ca^2+^ transients orchestrate a sequence of events involving the establishment of a block to polyspermy, liberation from metaphase II arrest, selective recruitment and degradation of maternal mRNA, pronuclear development, and initiation of embryonic gene expression. This process is collectively termed as “oocyte activation” and marks the transition from a gamete into an embryo. The nature, amplitude, duration, and frequency of the calcium oscillations are species-specific. In humans, this physiological signal lasts several hours and the profile of Ca^2+^ spikes appear to be related to embryo developmental competence [86–88]. Calcium imaging studies of eggs inseminated in vitro showed that the first wave of Ca^2+^ oscillations begins at sperm entry and spreads as a radial wave towards the opposite egg pole [89]. The finding that the injection of cytosolic sperm extract evokes characteristic Ca^2+^ oscillations implied that egg activation is triggered by a soluble factor delivered by the fertilizing spermatozoon [90–92]. It was hypothesized that the sperm-borne oocyte activation factor (SOAF) is released from perinuclear theca, a cytoskeletal coat over sperm head which dissolves concomitantly with sperm nuclear decondensation. SOAF then diffuses in ooplasm and triggers signaling cascade of oocyte activation via the coordinated release of Ca^2+^ from oocyte the endoplasmic reticulum stores. Interestingly, oocyte activation after ICSI occurs only when perinuclear theca of an injected spermatozoon is dissolved [93].

Various sperm proteins were evaluated for their ability to induce rises of [Ca^2+^]i and activate the egg [94–98]. Among them, the testes-specific isoform of phospholipase C, named PLC zeta, has been put forward as the strongest candidate for the long-sought-after soluble SOAF [99, 100]. Several lines of evidence from independent laboratories accumulated over the years supporting the contention that PLC zeta represents a physiological agent responsible for generating Ca^2+^ oscillations and triggering embryogenesis in mammals [87, 101]. This cytosolic sperm protein is introduced into the oocyte upon sperm entry, interacts with the yet-to-be-identified oocyte factor(s), and evokes the characteristic pattern of serial Ca^2+^ release in ooplasm [88]. Sperm with impaired expression of PLC zeta have reduced or absent capacity to elicit Ca^2+^ oscillations in the egg [102, 103]. The egg activation can be achieved in the absence of sperm by microinjection of mouse/human PLC zeta which induces fertilization-like Ca^2+^ signal and has the potential to trigger parthenogenetic embryo development [99, 100, 104–106]. Besides, cumulative clinical data suggests that PLC zeta deficiency contributes towards male and idiopathic infertility. Particularly cases with recurrent fertilization failure seem to be intimately linked with the function of PLC zeta [105, 107, 108].

The current tools to evaluate the fertilizing capacity of the sperm are restricted to the mouse oocyte activation test (MOAT). This test categorizes male patients according to the ability of their sperm to activate mouse oocyte development [109, 110]. When a high activation rate is detected by MOAT, the sperm-related problem is ruled out and oocyte factors are suspected to account for fertilization failure. However, a large volume of human oocyte represents a greater challenge for activation than a mouse oocyte. This is consistent with the finding that the human sperm contains a more potent variant of PLC zeta which can trigger Ca^2+^ oscillations in a lower concentration than the mouse enzyme [111]. It is thus possible that PLC zeta-deficient human spermatozoa, incapable of fertilizing human eggs, are still able to activate a smaller mouse oocyte. Due to the interspecies differences in the intrinsic activity of the key fertilization enzyme, the test employing heterologous mouse-human model has only limited diagnostic value. Besides, practical and legal restrictions concerning cross-species fertilization in many countries hinder the wide application of MOAT in clinical practice. There is much interest in developing a sensitive and robust prognostic assay for sperm activation potency. Implementing evaluation of PLC zeta function in clinical settings would enable appropriate management of the fertility treatment.

At present, assisted oocyte activation (AOA) is the only therapeutical option available for patients experiencing total fertilization failure following ICSI. However, in vitro treatment with calcium ionophores (e.g., calcimycin and ionomycin) does not induce repetitive Ca^2+^ oscillations. Instead, it leads to a steady increase of [Ca^2+^]i which might facilitate spontaneous activation in the absence of sperm. Moreover, exposure of the oocytes to both agents raises serious concerns regarding a long-term epigenetic effect of the non-physiological stimuli on the development of the resulting embryo [112]. Some research investigated the possibility of using a combination of ICSI with direct microinjection of PLC zeta protein or complementary RNA as an alternative to controversial AOA [106, 113]. Although current results are very promising, well-designed clinical studies are needed to confirm that PLC zeta indeed constitutes a “magic bullet” that would rescue the fertilization failure caused by the sperm factor before the method can be safely translated to clinical practice. Research should also focus on the molecular cascade triggered by PLC zeta following gamete fusion. It would be beneficial to identify the oocyte proteins contributing to activation deficiency as these molecules may also represent useful diagnostic and therapeutical targets [88].

### Block to polyspermy

**Fig. 2:**
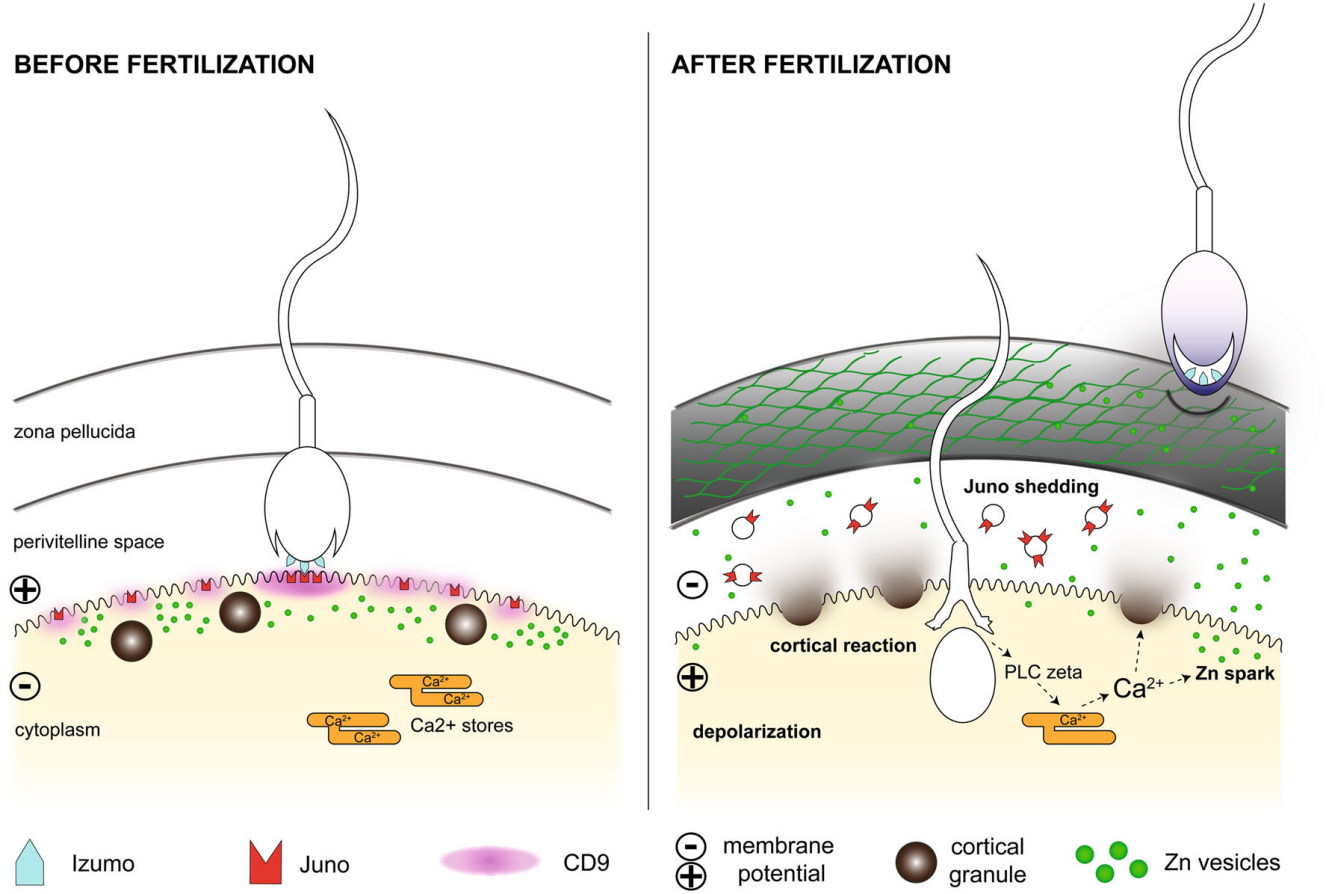
The mechanisms of prevention against polyspermy. Upon fertilization oocyte membrane becomes unreceptive to further sperm binding by 1) membrane depolarization and 2) removal of oocyte receptor Juno from oolemma. A permanent mechanical block is established with 3) cortical reaction and 4) Zn spark-induced hardening of the zona pellucida

The basic prerequisite for successful conception is that an egg is fertilized by a single sperm only. The presence of more than one male nucleus inside of the female gamete would cause severe genetic imbalance in the resulting embryo. To ensure monospermic fertilization, the oocytes evolved the series of safety mechanisms to avoid additional sperm fusion events, collectively referred to as a “polyspermy block” (Fig. 2). The entry of multiple spermatozoa is prevented by modification of the oolemma and egg vestments with the relative importance of these protective barriers varying among species [114].

#### Membrane depolarization

The membrane block to polyspermy was intensively studied in marine animals. The experiments showed that the attachment of the first sperm induces a rapid shift in the egg membrane potential from negative to positive level [115]. This alteration of the electric charge hinders other sperm attachment to the oocyte surface. This observation led to the conclusion that post-fertilization membrane depolarization represents a fast block to polyspermy. Although commonly referred to as an evolutionarily conserved safeguard response, the contribution of an electrically mediated membrane block is virtually unfounded in mammals, including humans. It is likely that here, the fast membrane depolarization is less important than in invertebrates and amphibians because the female reproductive tract limits arrival of the sperm to the fertilization site and the sperm-to-egg ratio is thus much lower than during external fertilization [116,^117^]. Besides, the membrane potential overshot is only transient, lasting up to several minutes. To establish an effective barrier to secondary fertilization, the short-acting electrical depolarization must be complemented by a permanent block.

#### Juno removal

An alternative mechanism for the establishment of a fast membrane block against polyspermy has been proposed. When investigating molecular details of the gamete fusion process in mice, Bianchi and colleagues observed that after the initial Izumo-Juno contact was established, remaining oocyte receptors were shed off the egg membrane within extracellular vesicles [76]. Rapid loss of Juno from the oocyte surface renders the already fertilized egg refractory to additional sperm binding. The authors speculated that the presence of the Juno-containing vesicles in the perivitelline space might also serve as a “decoy” which neutralizes incoming spermatozoa and prevents secondary fertilization. Interestingly, when gamete recognition was circumvented by ICSI, Juno was not removed from the oocyte surface and the egg remained receptive to further sperm [78]. This finding is consistent with previous observations that ICSI does not induce an effective block to polyspermy in mammalian eggs [118, 119]. Studies are being undertaken to provide the evidence that the removal of Juno from eggʼs surface contributes to the establishment of a polyspermy block in humans.

#### Cortical reaction

The most studied strategy on how to reduce the incidence of polyspermy is the slow mechanical block at the level of the eggʼs external coat. Once the egg is fertilized, the surrounding ZP becomes impermeable to late-coming sperm. Although molecular details of this process remain unclear, the crucial role of cortical granules (CGs) has been recognized. These membrane-bound secretory organelles are derived from the Golgi apparatus early in development [120] and during oocyte maturation, they progressively translocate towards the periphery in an actin-dependent manner [121]. In the periovulatory egg, the electron-dense CGs are densely aligned underneath the oocyte cortex, hence the name “cortical granules” [120, 122]. The oocyte activation following the first sperm penetration evokes Ca^2+^ oscillations which in turn trigger CG exocytosis, known as a “cortical reaction.” The fusion of CGs with the plasma membrane leads to a burst of their content into the perivitelline space. The released proteins cause a structural modification of ZP that slowly transforms the receptive outer coat of the oocyte to the hardened protective layer of the developing embryo [123]. Post-fertilization aging of the oocyte creates the risk that cortical reaction is triggered spontaneously without fertilization. Premature or partial exocytosis of CGs compromises the effectiveness of the ZP block and contributes to a high incidence of abnormal fertilization in aged oocytes [124].

Until recently, the content of CGs has been largely unknown. The identification of mouse ovastacin has shed light on mechanistic bases of the zona hardening process. This protease is discharged from CGs and cleaves glycoprotein ZP2, a building component of the ZP. The destruction of the primary sperm binding ligand in the zona matrix establishes a definite post-fertilization block to polyspermy. The role of ovastacin was supported by the finding that in ovastacin-deficient female mice, ZP2 remains intact after fertilization and sperm bind even to 2-cell stage embryos [125, 126]. Recent research revealed that the slow block to polyspermy is further regulated on a higher level. It has been shown that, in mouse oocytes, CG exocytosis is prevented by the abundance of a liver-derived plasma protein fetuin [127]. In 2013, Dietzel and colleagues took advantage of targeted gene depletion and mouse transplantation experiments to demonstrate that a member of the fetuin family, fetuin B, controls ovastacin activity. This study concluded that fetuin B is required to specifically inactivate traces of ovastacin constantly leaking from unfertilized oocytes [128]. In other words, the direct inhibition by fetuin-B restrains basal ovastacin activity and keeps the ovulated egg fertilizable. However, once a sperm has penetrated, the oocyteʼs cortical reaction is unleashed, and ovastacin surge will overwhelm the fetuin-B buffer capacity, thereby initiating the zona hardening. The finding that fertilization can be controlled by a liver-derived plasma protein demonstrates extreme complexity of the regulatory circuits orchestrating mammalian fecundity. Based on the mice data, the authors proposed that the addition of exogenous fetuin-B into the oocyte culture media might increase in vitro fertilization (IVF) success in humans [129]. So far, only two clinical studies assessed the association between the level of fetuin-B and fertilization rate in human IVF patients. Both showed that serum fetuin B level can serve as a predictive marker which may help to make an informed decision whether the oocytes should be fertilized by conventional IVF or ICSI that overcomes the ZP barrier [130, 131]. Moreover, it has been reported that pharmacological down-regulation of fetuin-B caused reversible infertility in female mice [132] suggesting that this blood protein might represent a molecular target for the development of novel therapies for short-term modulation of female fertility. Although the current experimental data is very promising, more clinical studies performed by independent research groups are warranted to confirm the role of fetuin-B human reproductive biology.

#### Zinc spark

During the last decade, zinc (Zn) has emerged as another essential element in ensuring monospermic fertilization. The work of Woodruff and colleagues has advanced our knowledge of the role of Zn in mammalian reproduction. In a series of experimental studies, the researchers used a combination of imaging and biochemical tools to study the Zn dynamics in mammalian oocytes before and after fertilization [133–139]. Selective Zn indicators revealed that intracellular Zn concentration extensively increases during oocyte maturation and its localization tracks show similar pattern to CGs staining. Accumulation of Zn is a physiological imperative to avoid a premature exit from meiosis and sustain metaphase II arrest. On the contrary, the rapid loss of Zn at the time of fertilization is required for the egg-to-embryo transition [134, 137]. Explosive Zn efflux immediately following sperm entry has been visualized by a vital fluorogenic zinc probe [136, 137]. Due to the characteristic radial glare pattern of an activated egg, this phenomenon has gained the name a “zinc spark.” Apart from being an early hallmark of successful fertilization, the serial exocytosis of Zn-containing vesicles contributes to the ZP hardening. The released metal ions execute their role differently than proteases stored in the CGs. Instead of cleaving glycoproteins of the ZP, exposure to Zn alters the architecture of the eggʼs outer layer by stabilizing glycoprotein chains and bundling fibril components of the extracellular matrix. Conformational change turns ZP into a stiff mechanical barrier that obstructs further sperm access to the already fertilized egg [138]. The massive increase of extracellular Zn concentration establishes a “zinc shield” which could derail Zn signaling in late-coming spermatozoa thus adding a further component to anti-polyspermy defense [140].

The fertilization-induced zinc spark appears to be evolutionary conserved across mammalian species [133, 136, 137]. In mice, the amount of Zn released at the time of fertilization correlates with successful embryo development to the blastocyst stage [136]. This finding raises the intriguing option to develop the platform to quantify the zinc spark in humans. Non-invasive measurement of extracellular Zn in the culture medium would enable prospective selection of the best quality embryos for fertility treatment. Besides, the early detection of activation failure in oocytes conventionally inseminated in vitro would leave the opportunity to perform rescue ICSI during routine working hours. This approach would aid in the prevention of the overuse of ICSI in clinical practice. Furthermore, experimental treatment with Zn-specific chelator demonstrated that reduction of intracellular Zn is sufficient to induce human egg activation [137]. As shown in the porcine system, this kind of treatment may increase the efficacy of AOA, particularly in calcium ionophore-resistant cases [141, 142]. New findings regarding the Zn role in reproduction should be taken into consideration in search of optimal conditions for in vitro maturation (IVM) of human oocytes. It has been shown that supplementation of IVM media with adequate Zn concentration improved maturation and developmental potential of porcine oocytes. It would be interesting to examine if such treatment could also enhance cytoplasmic maturation and fertilization rate of oocytes in human IVM cycles [143].

There are still many unanswered questions regarding strategies mammalian eggs use to reduce the risk of multiple sperm penetration. Future studies might bring new revelations showing that the process is more complex than previously acknowledged. Yet, the importance of the polyspermy block in mammals is being disputed by some researchers who are raising concerns regarding the relevance of research data from in vitro inseminations. They point out that, in vivo, the female reproduction tract greatly limits the number of sperm reaching the fertilization site, thus reducing the risk of simultaneous fertilization. But, under laboratory conditions, high rates of polyspermy are artificially induced by increasing the sperm concentration [144]. Therefore, the experiments involving oocytes deprived of their vestments or exposed to an unnaturally high number of spermatozoa might be unrepresentative for the physiological situation and must be interpreted with caution [117].

Although most embryos inheriting an extra set of chromosomes die during the cleavage stage, some can give rise to a viable blastocyst and survive into gestation. Based on cytogenetic studies performed on spontaneous abortions, polyspermy is estimated to account for 2–3% of natural pregnancies [114, 145–148]. However, the actual incidence of this phenomenon might be underreported since, in vivo, arrest in preimplantation development, implantation failure, and early pregnancy loss goes unnoticed. Interestingly, some cases of atypical twinning have been attributed to the dispermic fertilization [149–151]. Detailed genotyping implicated the double sperm penetration in the case of live-born monochorionic but sex-discordant twins. The pair shared 78% of their parental genome while maternally derived genetic information was identical. This “sesquizygosity” is thought to arise from heterogoneic assortment of paternal genomes following fertilization of a single egg by two spermatozoa [151].

### Future perspectives

Conception is the most intimate moment of our lives. Although being of great scientific and clinical relevance, understanding of the molecular basis of fertilization remains rudimentary. Most of what we know about sperm-oocyte interaction stems from animal studies. The development of IVF methods offered a simplified functional assay to study mechanistic and molecular aspects of the mammalian fertilization in laboratory conditions. Furthermore, the ability to create gene-deficient “knockout” mice permitted us to assess the in vivo relevance of molecules implicated in gamete recognition [47]. However, extrapolating research results from animals to humans is problematic due to interspecific differences in reproduction and fertility. The paucity of scientific knowledge about human fertilization is largely attributed to a unique nature and heterogeneity of gametes which are extremely difficult to be experimentally manipulated. The procurement of human oocytes for research purposes is limited to only a small number of surplus oocytes rejected for fertility treatment. Apart from that, the research that brings human eggs and sperm together in the same dish is forbidden in most countries. The stakeholders’ agreement of ethical acceptance of experiments involving coincubation of human gametes would stimulate the research in this field. Alternatively, development of fully functional human gametes in vitro using genetic reprogramming or differentiation from human embryonic stem cells would help to bypass ethical and legal restrictions [152].

There are powerful genomic, imaging, and analytical tools available to study the delicate sperm-egg dialogue. Technical advancements now enable in-depth whole-genome, transcriptome, proteome, and secretome profiling of single cells. Until recently, the loss-of-function assays in human gametes were virtually impossible. However, the revolutionary gene-editing technique opens a new area of research allowing researchers to dissect the role of individual molecules in the process of gamete recognition and fusion [153]. The recently developed Trim-Away method for acute and rapid degradation of endogenous proteins provides the opportunity to assess the requirement of a selected molecular candidate for fertilization competence in human oocytes [154, 155]. These targeting approaches offer an elegant alternative to immunological techniques and non-specific pharmacological interventions. In addition, the potential of cell-penetrating peptides (CPP) to deliver selected bioactive moieties across the plasma membrane has been investigated in various cells, including bovine and human sperm [156, 157]. The experimental data suggest that CPP can traffic small regulatory proteins and thus directly influence sperm physiology including progesterone-induced hyperactivation. The idea to temporarily halt sperm motility either by CPP or small-molecular inhibitors interfering with the CatSper function is very appealing to the pharmaceutical industry. However, controlling millions of sperm poses an enormous challenge as opposed to the ovulation of a single egg. Clinical trials assessing efficiency and safety are essential before the concept of a male pill can be marketed. The search for birth control agents centers on the Juno and Izumo interaction. The crystallographic study which determined structure of these receptors represents a necessary stepping stone towards a rational design of a drug which would act as a short-acting non-hormonal contraceptive [158].

There is another good reason to investigate the sex cell interaction. Unveiling the secrets of human fertilization might bring new therapeutic options for millions of patients suffering from infertility. The identification of molecules that are pivotal for sperm functionality and interaction with the egg paves the way for the development of innovative diagnostic tools and biomarker-based assays for the selection of fertilization-competent gametes in human IVF. Although the translation of scientific discoveries to improved healthcare is generally slow, current findings might soon find practical application in reproductive medicine. Particularly the combination the functional tests with microfluidics holds potential to simulate natural selection occurring in vivo. By increasing our knowledge of human procreation, in the future, certain fertility issues may be treatable or even cured, for example, by stimulating relevant signaling pathways or by genetic therapy, rather than bypassing them with ICSI.
